# Induction of PI3K/Akt-Mediated Apoptosis in Osteoclasts Is a Key Approach for *Buxue Tongluo* Pills to Treat Osteonecrosis of the Femoral Head

**DOI:** 10.3389/fphar.2021.729909

**Published:** 2021-11-29

**Authors:** Dan Wang, Yicheng Liu, Dandan Tang, Shujun Wei, Jiayi Sun, Lvqiang Ruan, Lin He, Ruolan Li, Qiang Ren, Xiaoping Tian, Yunhui Chen

**Affiliations:** ^1^ School of Pharmacy, School of Basic Medicine, Innovative Institute of Chinese Medicine and Pharmacy, School of Acupuncture and Tuina, Chengdu University of Traditional Chinese Medicine, Chengdu, China; ^2^ Neijiang Hospital of Traditional Chinese Medicine Affiliated to Chengdu University of Traditional Chinese Medicine, Neijiang, China; ^3^ Hospital of Chengdu University of Traditional Chinese Medicine, Chengdu, China

**Keywords:** buxue tongluo pill, osteonecrosis of the femoral head, GEO database, apoptosis, PI3K/AKT

## Abstract

The *Buxue Tongluo* pill (BTP) is a self-made pill with the functions of nourishing blood, promoting blood circulation, dredging collaterals, and relieving pain. It consists of *Angelica sinensis* (Oliv.) Diels, *Pheretima aspergillum* (E.Perrier), *Panax notoginseng* (Burk.) F. H. Chen, *Astragalus membranaceus* (Fisch.) Bge, and *Glycyrrhiza uralensis* Fisch. Various clinical practices have confirmed the therapeutic effect of BTP on osteonecrosis of the femoral head (ONFH), but little attention has been paid to the study of its bioactive ingredients and related mechanisms of action. In this study, UPLC/MS-MS combined with GEO data mining was used to construct a bioactive ingredient library of BTP and a differentially expressed gene (DEG) library for ONFH. Subsequently, Cytoscape (3.7.2) software was used to analyze the protein–protein interaction between BTP and DEGs of ONFH to screen the key targets, and functional annotation analysis and pathway enrichment analysis were carried out. Finally, 34 bioactive compounds were screened, which acted on 1,232 targets. A total of 178 DEGs were collected, and 17 key genes were obtained after two screenings. By bioinformatics annotation on these key genes, a total of 354 gene ontology (GO) functional annotation analyses and 42 Kyoto Encyclopedia of Genes and Genomes (KEGG) pathways were obtained. The present study found that GO and KEGG enrichment were mainly related to apoptosis, suggesting that BTP may exert an anti-ONFH effect by promoting osteoclast apoptosis. Experiments *in vitro* demonstrated that BTP could increase the mitochondrial membrane potential (MMP) and induce remarkable apoptosis in osteoclasts. Furthermore, we determined the apoptosis marker of cleaved(C)-caspase-3, bcl-2, and bax and found that BTP could upregulate the C-caspase-3 and bax expression in osteoclasts and decrease the expression of bcl-2, p-Akt, and p-PI3K in a dose-dependent manner, indicating that BTP could induce PI3K/Akt-mediated apoptosis in osteoclasts to treat ONFH. This study explored the pharmacodynamic basis and mechanism of BTP against ONFH from the perspective of systemic pharmacology, laying a foundation for further elucidating the therapeutic effects of BTP against ONFH.

## Introduction

Osteonecrosis of the femoral head (ONFH) refers to a disease in which the blood supply of the femoral head is damaged or interrupted, leading to the death of bone marrow components and bone cells and the structural change and collapse of the femoral head (Pouya and Kerachian, 2015). It has also been considered a debilitating disease of multifactorial genesis, predominately affecting young people aged 20–40 years with the destruction of hip joints in their third, fourth, or fifth decade of life (Sun and Li, 2013). Currently, approximately 20,000–30,000 patients in the United States are diagnosed with ONFH annually, bringing pain and a substantial economic burden to the patients. Treatment options available for ONFH include pharmaceutical intervention and surgical treatment. Unfortunately, the former often fails to achieve satisfactory results due to the rapid progress of ONFH, and concerns regarding the longevity of the hip replacement have always existed. Thus, ONFH has been widely regarded as a global problem (Ping, 2021). Recently, traditional Chinese medicine (TCM) has been gradually accepted as a complementary and alternative medicine for the treatment of multiple diseases due to low toxicity and good curative effects (Xu et al., 2021; Zhang et al., 2021). A growing amount of TCM compounds and monomers, such as *Bushen Huoxue* decoction (Sun and Li, 2013), *Huangqi Shenggu* decoction (Ping, 2021), *Taohong Siwu* decoction (Zhao, 2020), Gastrodin (Zheng et al., 2014), and luteolin (Yan et al., 2020), have been reported to exert a significant therapeutic effect on ONFH.

**FIGURE 1:**
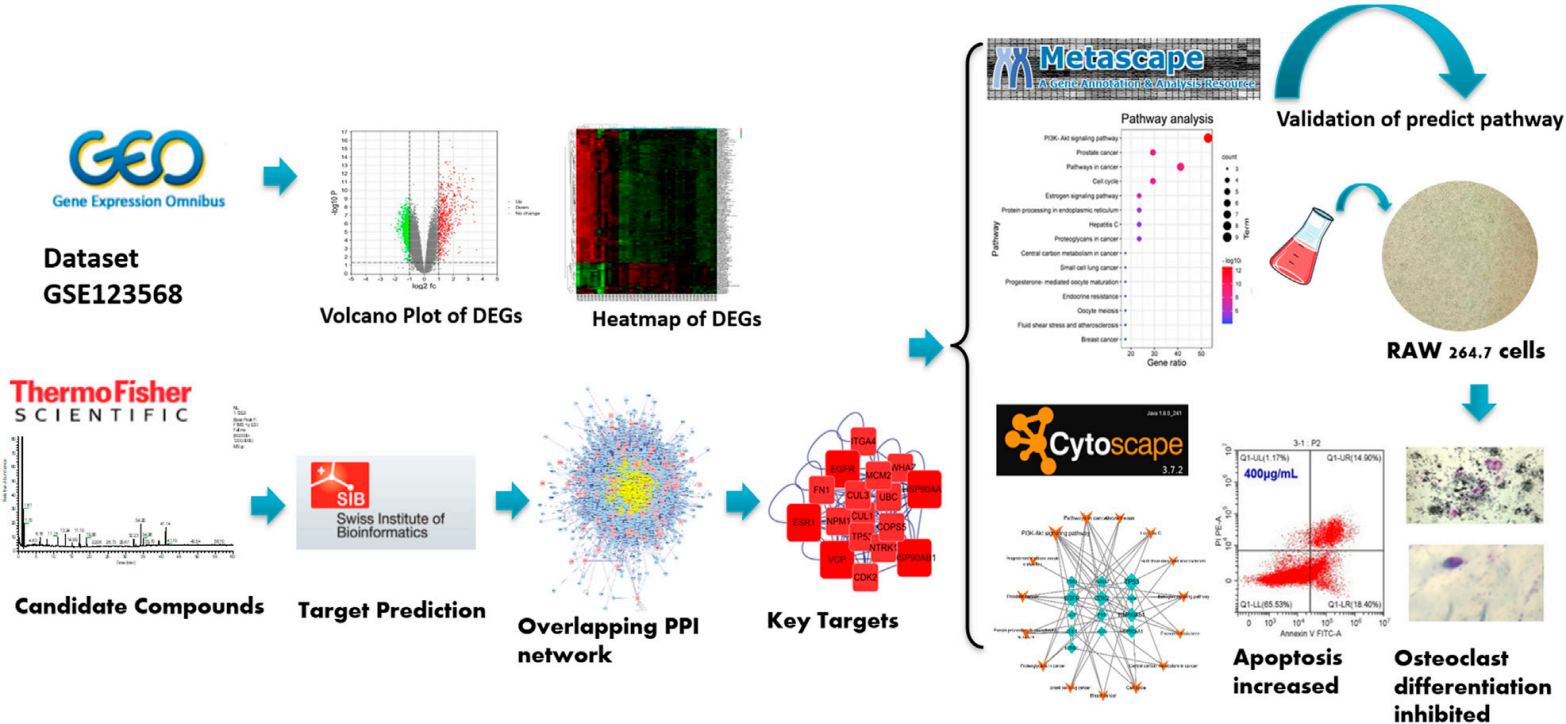
Whole experiment process in our study.

BTP is a TCM formula that has been proven effective in treating ONFH with the functions of “promoting blood supply”. It is composed of *Angelica sinensis* (Oliv.) Diels, *Pheretima aspergillum* (E.Perrier), *Panax notoginseng* (Burk.) F. H. Chen, *Astragalus membranaceus* (Fisch.) Bge, and *Glycyrrhiza uralensis* Fisch. Although it is frequently used to treat ONFH in clinical practice, there is no research for the specific mechanism regarding the anti-ONFH effect of BTP. Moreover, the TCM formula is characteristic of multicomponents, multitargets, and multipathways, making it difficult to reveal their mechanisms of action (Xu et al., 2021). Network pharmacology is a technology that can systematically analyze the interaction network of drug components, targets, diseases, and genes, which is consistent with the overall concept of the TCM theoretical system (Hopkins, 2008). At present, increasing studies have confirmed the great potential of network pharmacology in investigating the possible molecular mechanisms of TCM (Shan et al., 2018; Cui et al., 2018; Daina et al., 2019; Wang et al., 2020; Xia et al., 2021). Thus, it is an inevitable trend to apply network pharmacology to investigate the material basis and molecular mechanism of the TCM formula (Qing and Jia, 2021; Qing and Hu, 2021; Qing and Jia, 2019). In the current study, UPLC-MS/MS was used to identify the active ingredients of BTP, and network pharmacology was used to study the molecular mechanisms of BTP against ONFH. Besides, experiments *in vitro* were performed to provide scientific evidence for the predicted results with the network pharmacology. The general experimental procedures are shown in Figure 1.

## Methods

### Preparation of the Extracts of BTP

BTP samples were prepared and provided by Neijiang traditional Chinese medicine hospital (Neijiang, China). The powder of BTP (10 g) was ultrasonically dissolved with 300 ml, 75% ethanol, followed by concentrating to 100 ml using a rotary evaporator. Then, the supernatant was filtered by microporous membranes with a pore size of 0.22 μm. Then, the obtained 10 μl filtrates of BTP were analyzed by UPLC-MS/MS.

### UPLC-MS/MS Analysis

For qualitative analysis, a Thermo Scientific Q Exactive Orbitrap HRMS (Thermo Fisher Scientific, Massachusetts, United States) was connected to a Thermo Scientific Vanquish UPLC (Thermo Fisher Scientific, Massachusetts, United States). Chromatographic separation was achieved on a Thermo Scientific Accucore C_18_ (3 × 100 mm, 2.6 μm) in a thermostatically controlled column compartment (30 °C). The aqueous and organic mobile phases used were acetonitrile (A) and 0.1% phosphoric acid aqueous solution (B), respectively. The gradient elution program was set up as follows: 0–20 min, 12–25% A; 20.1–30 min, 25–28% A; 30.1–32 min, 28–40% A; 32.1–40 min, 40–50% A; 40.1–60 min, 50% A; 60.1–65 min, 12% A. The flow rate was set as 0.3 ml/min, and 2 μl of the extraction was injected into the UPLC system. The instrument was operated in the positive ion mode to perform full-scan analysis over an m/z range of 100–1,500. The optimized parameters were set as follows: a sheath gas flow rate of 35 L/min; a spray voltage of 3000 V; a capillary temperature of 320 V; an aux gas flow rate of 10.00 L/min; a max spray current of 100 A; a probe heater temperature of 350 C; and an S-lens RF level of 50.00%.

### Establishment of Effective Compounds and the Target Library of BTP

Compounds of BTP were analyzed by UPLC-MS/MS. Subsequently, targets regarding these identified compounds via UPLC-MS/MS were obtained from the online database of SwissTargetPrediction (http://www.swisstargetprediction.ch/) (Daina et al., 2019).

### Establishment of the ONFH Target Library

As a common worldwide resource for gene expression studies, the Gene Expression Omnibus (GEO) database (https://www.ncbi.nlm.nih.gov/geo) allows open access to high-throughput gene expression and other functional genomics datasets (Clough and Barrett, 2016). The GEO database was searched using “osteonecrosis of femoral head” as the keyword. We aimed to find a comparison of gene expression data sets between femoral head samples from patients with ONFH and normal femoral head samples. In order to ensure the stability and credibility of the analysis results, only data sets with a sample size larger than 10 were selected. Finally, only one suitable data set of ONFH was downloaded from the GEO database, namely, dataset GSE123568. In this dataset, 10 normal femoral head samples and 30 ONFH samples were included.

### Acquisition of Differentially Expressed Genes

In order to obtain DEGs, dataset GSE123568 was analyzed with GEO2R, which was the official differential expression analysis tool of the GEO database. In this study, the difference with logFC (fold change) ≥1.3 and *p*-values <0.01 was statistically significant, and the corresponding genes were identified as DEGs.

### Construction of the Protein–Protein Interaction Based on Bisogenet

In this study, the protein–protein interaction (PPI) of BTP and DEGs from ONFH were constructed by Cytoscape software (3.7.2). Then, the overlapping PPI network was obtained by using BisoGenet in Cytoscape. The median of three important attribute values, namely, degree, betweenness, and closeness, was employed for screening to obtain the core gene. After two screenings, a PPI network containing 17 nodes and 114 edges was obtained and used for enrichment analysis. The detailed attribute values and screening process of these targets are presented in Table 3 and Figure 3, respectively.

### Functional Enrichment and Constructing the Network of BTP-DEGs-Pathwa*y*

The Metascape platform (http://metascape.org/gp/index.html) has a comprehensive annotation function and data of gene annotation monthly updated (Zhou et al., 2019). In this study, functional annotation of gene ontology (GO) and KEGG pathways was performed with the Metascape platform. The main biological processes and involved metabolic pathways were analyzed by submitting the core targets of BTP-DEGs from ONFH to Metascape with *p* < 0.01. Then, the results were saved and visualized by Bioinformatics (http://www.bioinformatics.com.cn/) (Huang, 2009). Afterward, as shown in Figure 4, the network of the BTP-DEGs-Pathway was constructed with Cytoscape (3.7.2).

## Experimental Validation

### Chemicals and Reagents

Fetal bovine serum (FBS), phosphate-buffered saline (PBS), penicillin–streptomycin, 0.25% trypsin–EDTA (1x), and Dulbecco’s modified eagle’s medium (DMEM) were purchased from GIBCO (Grand Island, NY, United States). A cell counting kit-8 (CCK8) detection kit was purchased from the Beijing 4A Biotech Co., Ltd. (Beijing, China). The assay kits for DCFH-DA, horseradish peroxidase (HPR)-conjugated secondary antibodies, β-actin, BCA protein assay reagents, and primary antibodies for cleaved (C) caspase-3 were purchased from Boster Biol. Tech. (Wuhan, China). Primary antibodies for phosphorylation- (p-) Akt, Akt, PI3K, and P-PI3K were obtained from ImmunoWay Biotechnology Co. (Suzhou, China). Annexin V-FITC/PI apoptosis kits were obtained from US Everbright^®^ Inc (Suzhou, China). Polyvinylidene fluoride (PVDF) membranes were purchased from Sigma-Aldrich (Shanghai, China). 5,5′,6,6′-Tetrachloro-1.1′,3.3′-tetraethyl-imidacarbocyanine iodide (JC-1) was obtained from Jiangsu KeyGen Biotech (Nanjing, China).

### Cell Culture

The RAW 264.7 cells were purchased from Wuhan Pu-nuo-sai Life Technology Co., Ltd. (Wuhan, China) and used throughout the study. The RAW264.7 cells were cultured in DMEM containing 10% FBS (v/v) and 1% penicillin–streptomycin (v/v) at 37°C in a humidified atmosphere of 5% CO_2_.

### Cell Activity Detection

The RAW 264.7 cells were incubated in 96-well plates (1×10^4^ cells/well) with different concentrations (0, 5, 10, 20, 40, 60, 80, 100, 150, and 200 μg/ml) of BTP, followed by induction with RANKL (100 ng/ml). After incubation for 24 and 48 h, the supernatant was discarded, and the cells were washed three times with PBS. Then, 10 µl CCK-8 was added to each well and incubated for 0.5 h. The optical density (OD) values were measured at 450 nm by the microtablet reader (Bio-RAD, United States). Each experiment was repeated three times. The concentration- and time-dependent effects of BTP on raw 264.7 cells are presented in Figure 5.

### Osteoclastogenesis and TRAP Staining

The RAW 264.7 cells were seeded in 24-well plates (1×10^5^ cells/well) and then pretreated with BTP (0, 50, 75, and 100 μg/ml) for 24 h, followed by induction with RANKL (100 ng/ml) for 7 days. Untreated cells were used as a negative control, while only RANKL-induced cells were used as a positive control group. During the induction period, the medium was changed every other day. After 7 days, the cells were washed with PBS, followed by fixing with 4% paraformaldehyde for 30 min, and then stained to detect TRAP activity. Finally, trap-positive cells were enumerated via a microscope. Mature osteoclasts with more than three nuclei were counted in a random area in each replicate sample.

### Bone Resorption Assay

The RAW 264.7 cells were seeded in 96-well plates (1×10^4^ cells/well) containing bovine bone slices and then pretreated with different concentrations of BTP (0, 50, 75, and 100 μg/ml) for 24 h, followed by induction with RANKL (100 ng/ml) for 9 days. Then, bovine bone slices seeded with cells were washed with PBS, fixed in 2.5% glutaraldehyde over 10 min, ultrasonically cleaned with 0.25 mol/l ammonia hydroxide, and stained with 1% toluidine blue over 10 min. After washing with ddH2O, the osteolysis pits were photographed with a light microscope. Bone resorption capacity was defined as the bone resorption relative area (resorption area/total bone area).

### Apoptosis Assay Using a Flow Cytometer

Cell apoptosis was detected by flow cytometry (CytoFLEX FCM, Beckman Coulter Inc., Atlanta, Georgia, United States). The RAW 264.7 cells were incubated in 6-well plates with RANKL (100 ng/ml) for 5 days and then treated with BTP (0, 50, 75, and 100 μg/ml) for 24 h. Subsequently, cell apoptosis was detected with the manufacturer’s instructions of the Annexin V/PI apoptosis kit and cell cycle kit; the cells were stained with Annexin V-FITC and PI, respectively, and then detected by flow cytometer analysis. Each experiment was repeated three times.

### Mitochondrial Membrane Potential (MMP) Determination

The change of intracellular mitochondrial membrane potential (MMP, ΔΨm) is generally recognized as an important indicator of mitochondrial dysfunction, and JC-1 is commonly considered as an ideal probe to evaluate ΔΨm. In this study, the cells were inoculated in laser confocal dishes (1×10^4^/well), treated with different concentrations of BTP for 24 h, and subsequently induced with RANKL (100 ng/ml) for 5 days. After the induction, the cells were incubated with the JC-1 probe (10 μg/ml) in the dark for 30 min. Confocal laser microscopy was used to observe the green and the red fluorescence (Leica, SP8 SR, Wetzlar, Germany) of cells at 488 and 647 nm, respectively, and the images were recorded.

### Immunofluorescence Assay of AKT/P-AKT

The RAW 264.7 cells were seeded in the confocal dish (1×10^5^) and processed as described above. Then, they were washed three times with PBS, fixed with 4% paraformaldehyde for 30 min, and then washed three times with PBS. Afterward, the cells were treated with 0.5% Triton-X-100 for 30 min, followed by blocking with goat serum for 1 h at room temperature, and then, PI3K and P-PI3K antibodies (1:500) were added separately and incubated overnight at 4°C. Soon afterward, antirabbit IgG (H&L) Alexa Fluor^®^ 488 and antimouse IgG (H + L) Alexa Fluor^®^ 647 were added and incubated at room temperature for 1 h in the dark. Then, PBS was used to wash out the unbound secondary antibody, and the nuclei were stained with DAPI. Then, AKT/P-AKT and their fluorescence intensity were observed using a confocal laser microscope (Leica, SP8 SR, Wetzlar, Germany).

### Western Blotting Assay

The RAW 264.7 cells were inoculated in 6-well plates (2×10^6^/well) at 37°C in a humidified atmosphere of 5% CO_2_ and incubated for 24 h. Then, the RAW 264.7 cells were treated with RANKL (100 ng/ml) for 5 days and then treated with BTP (50, 75, and 100 μg/ml) for 24 h. At the end of the treatment, 100 μl of RIPA cell lysates was used to extract the total cell protein. The protein concentrations were determined using BCA protein assay reagents. Then, the total protein was separated by 12% SDS-PAGE and then transferred to the PVDF membrane. After blocking by 5% skimmed milk, the PVDF membrane was incubated overnight with diluted primary antibodies of PI3K, P-PI3K, C-caspase-3, Akt, and p-Akt (dilution 1:1,000) at 4°C. Subsequently, the PVDF membrane was incubated for 1 h with the diluted secondary antibody. Finally, the protein bands were stained with ECL detection kits, and β-actin was used as the internal reference. Image analysis software ImageJ (version 1.51, National Institutes of Health, MD, United States) was utilized for gray analysis.

### Statistical Analysis

All the data were presented as the mean ± standard deviations (SDs), statistical comparisons were evaluated using one-way analysis of variance (ANOVA), and significant differences between the mean values were measured using Duncan’s multiple range test. *p* < 0.01 was deemed statistically significant.

## Results

### Effective Compounds of BTP

**FIGURE 2:**
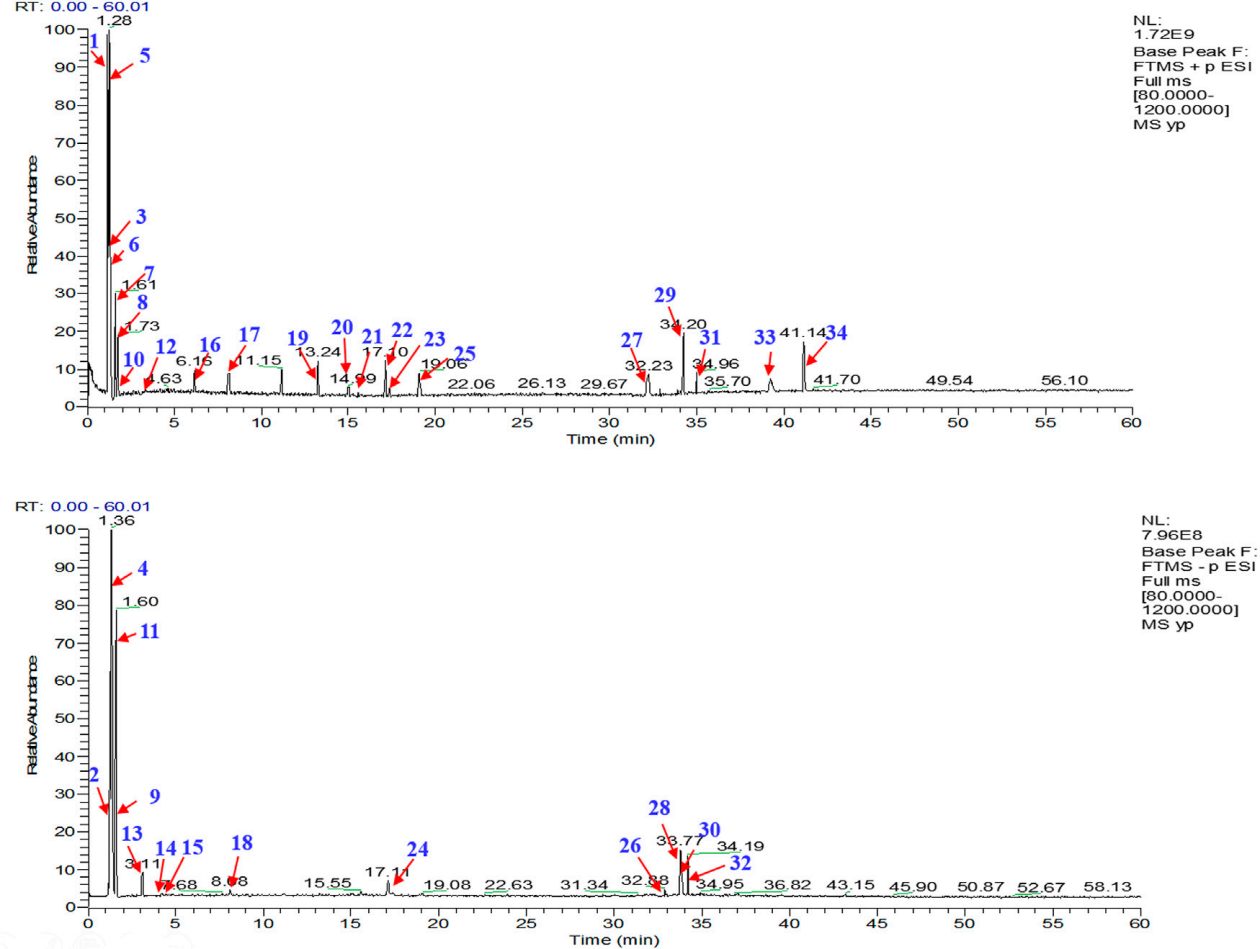
Total ion current diagram of BTP with positive **(upper)** and negative modes **(lower)**.

A total of 34 components were identified from BTP, of which six compounds belonged to *Angelica sinensis* (Oliv.) Diels, seven to *Astragalus membranaceus* (Fisch.) Bge, six to *Glycyrrhiza uralensis* Fisch., six to *Panax notoginseng* (Burk.) F. H. Chen, and nine to *Pheretima aspergillum* (E.Perrier). Their specific information and total ion current diagram are shown in Table 1, Table 2, and Figure 2, respectively. Moreover, a total of 1,232 related targets were obtained from the online databases.

**TABLE 1:** Precursor and product ions of constituents in the *Buxue Tongluo* Pill.

No.	Compound	RT	Formula	MS^1^	MS^2^	Herbal medicine	Ref.
1	dl-Arginine	1.19	C_6_H_14_N_4_O_2_	175 [M + H]^+^	116, 71, 70, 60	*P. aspergillum*	(Zhang et al., 2017)
2	Asparagine	1.23	C_4_H_8_N_2_O_3_	131 [M-H]^-^	114, 113, 95, 72	*A. membranaceus*	(Liu et al., 2013)
3	Choline	1.24	C_5_H_13_NO	104 [M + H]^+^	60	*A. membranaceus*	(Wu et al., 2020)
4	Sucrose	1.25	C_12_H_22_O_11_	729 [2M + FA-H]^-^	683.625, 387.11475, 179.89	*A. membranaceus*	(Wu et al., 2020)
5	Adenine	1.26	C_5_H_5_N_5_	136 [M + H]^+^	119, 109	*P. aspergillum*	(Wu et al., 2020)
6	Guanine	1.33	C_5_H_5_N_5_O	152 [M + H]^+^	135, 110, 81, 55	*P. aspergillum*	(Zhang et al., 2017)
7	Guanosine	1.33	C_10_H_13_N_5_O_5_	284 [M + H]^+^	152, 135,110	*P. aspergillum*	(Zhang et al., 2017)
8	Hypoxanthine	1.34	C_5_H_4_N_4_O	137 [M + H]^+^	119, 110	*P. aspergillum*	(Zhang et al., 2017)
9	Nicotinic acid	1.37	C_6_H_5_NO_2_	124 [M-H]^-^	96, 80, 78, 52	*P. aspergillum*	(Zhang et al., 2017)
10	Phenylalanine	1.41	C_9_H_11_NO_2_	166 [M + H]^+^	120, 103	*P. aspergillum*	(Zhang et al., 2017)
11	Succinic acid	1.46	C_4_H_6_O_4_	117 [M-H]^−^	99, 73	*P. aspergillum*	(Zhang et al., 2017)
12	Tryptophan	2.44	C_11_H_12_N_2_O_2_	205 [M + H]^+^	188, 170, 159, 142, 132, 130, 118	*P. aspergillum*	(Zhang et al., 2017)
13	Caffeic acid	3.01	C_9_H_8_O_4_	179 [M-H]^−^	135	*A. sinensis*/*P. notoginseng*	(Liu et al., 2017)
14	Benzoic acid	5.19	C_7_H_6_O_2_	121 [M-H]^−^	93	*A. sinensis*	(Liu et al., 2017)
15	3-Coumaric acid	6.47	C_9_H_8_O_3_	163 [M-H]^-^	119, 93	*P. notoginseng*	(Liu et al., 2019)
16	Calycosin-7-O-β-D-glucoside	7.37	C_22_H_22_O_10_	447 [M + Na]^+^	285, 270	*A. membranaceus*	(Zhang et al., 2008)
17	Chlorogenic acid	8.08	C_16_H_18_O_9_	355.10 [M + H]^+^	191, 179, 173	*A. sinensis*	(Liu et al., 2017)
18	Liquiritin	8.12	C_21_H_22_O_9_	417 [M-H]^-^	255, 135, 119	*G. uralensis*	[Meng et al., 2010]
19	Ferulic acid	13.24	C_10_H_10_O_4_	195 [M + H]^+^	177, 145	*A. sinensis*	(Chen et al., 2009)
20	Isoliquiritin	14.99	C_21_H_22_O_9_	419 [M + H]^+^	257, 147, 137	*G. uralensis*	(Zhang et al., 2011)
21	Daidzein	15.31	C_15_H_10_O_4_	255 [M + H]^+^	137.91	*A. membranaceus*	[Meng et al., 2010]
22	Ononin	17.11	C_22_H_22_O_9_	431 [M + H]^+^	268	*A. membranaceus*	(Luo et al., 2020)
23	Formononetin	17.34	C_16_H_12_O_4_	269 [M + H]^+^	254, 237,137	*A. membranaceus*	(Luo et al., 2020)
24	Ginsenoside Re	17.41	C_48_H_82_O_18_	991 [M-H]^-^	789, 441	*P. notoginseng*	(Liu et al., 2004)
25	Biochanin A	19.06	C_16_H_12_O_5_	285 [M + H]^+^	270, 253	*A. membranaceus*	(Luo et al., 2020)
26	Licoricesaponin G2	31.46	C_42_H_62_O_17_	837 [M-H]^-^	351,113	*G. uralensis*	(Meng et al., 2009)
27	Psoralen	32.23	C_11_ H_6_ O_3_	186 [M + H]^+^	187.03, 131.04	*P. notoginseng*	(Yang et al., 2010)
28	Ginsenoside Rb_1_	33.90	C_54_H_92_O_23_	1,107.59 [M-2H]^2-^	945.54, 783.48, 621.43	*P. notoginseng*	(Liu et al., 2004)
29	Pimpinellin	34.20	C_13_H_10_O_5_	247 [M + H]^+^	231,217,203,186,176	*A. membranaceus*	(Liu et al., 2012)
30	Gypenoside XVII	34.27	C_48_H_82_O_18_	991 [M-H]^-^	945.54, 783.48, 621.43	*P. notoginseng*	(Jia et al., 2019)
31	Isoliquiritigenin	34.96	C_15_H_12_O_4_	257 [M + H]^+^	147, 137	*G. uralensis*	(Zhao et al., 2016)
32	Glycyrrhizic acid	34.97	C_42_H_62_ O_16_	821 [M-H]^-^	645, 351	*G. uralensis*	(Liu et al., 2004)
33	Licochalcone A	39.42	C_21_H_22_O_4_	339 [M + H]^+^	297, 121	*G. uralensis*	(Zhang et al., 2011)
34	Z-Ligustilide	41.13	C_12_H_14_O_2_	232.13 [M + ACN + H]^+^	191, 173	*A. sinensis*	(Sheng et al., 2020)

MW, molecular weight, C7DG, calycosin-7-O-β-D-glucoside.

**TABLE 2:** Active compounds of the *Buxue Tongluo* Pill.

Id	Molid	Name	Structure
DG1	MOL000414	Caffeic acid	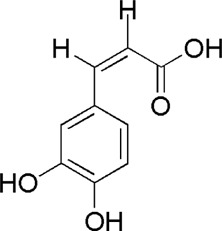
DG2	MOL004781	Pimpinellin	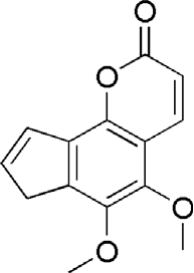
DG3	MOL002122	Z-Ligustilide	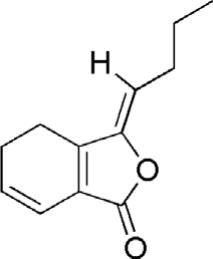
DG4	MOL000360	Ferulic acid	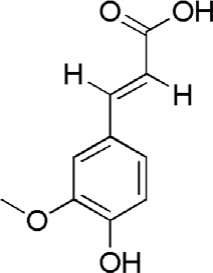
DG5	MOL001955	Chlorogenic acid	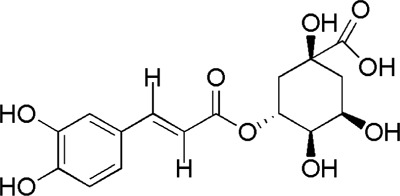
DG6	MOL000219	Benzoic acid	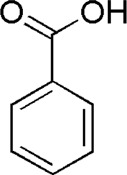
HQ1	MOL001788	Adenine	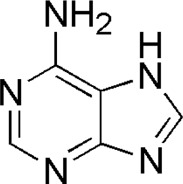
HQ2	MOL000391	Ononin	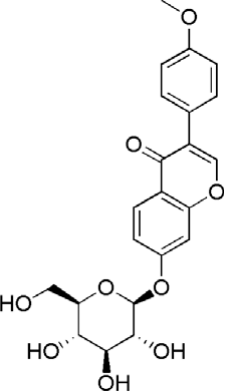
HQ3	MOL000510	Biochanin A	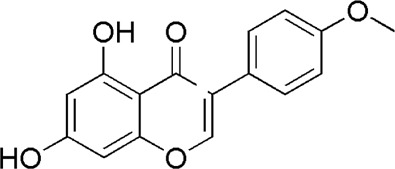
HQ4	MOL000392	Formononetin	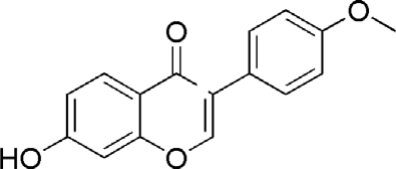
HQ5	MOL000842	Sucrose	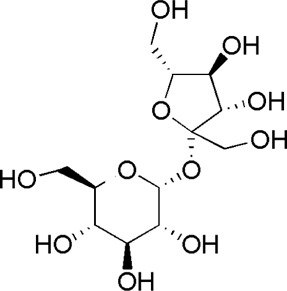
HQ6	MOL009290	Calycosin-7-O-β-D-glucoside	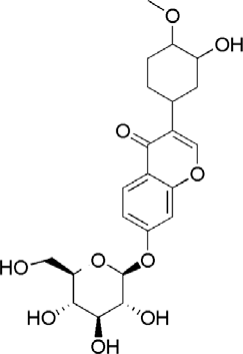
HQ7	MOL000429	Asparagine	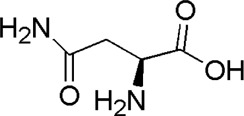
HQ8	MOL000390	Daidzein	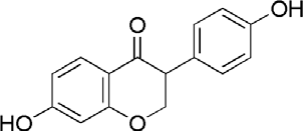
DL1	MOL000346	Succinic acid	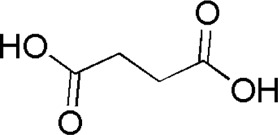
DL2	MOL001831	Hypoxanthine	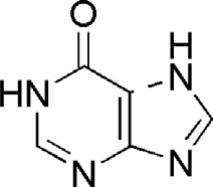
DL3	MOL000054	dl-Arginine	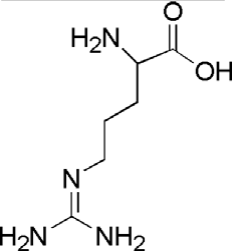
DL4	MOL000421	Nicotinic acid	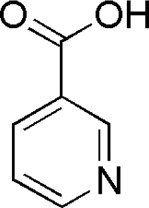
DL5	MOL001757	Guanine	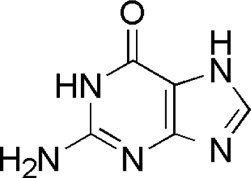
DL6	MOL002687	Guanosine	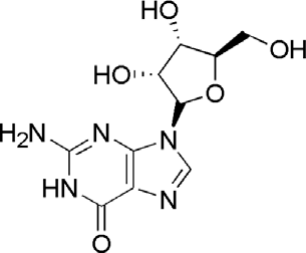
DL7	MOL000041	Phenylalanine	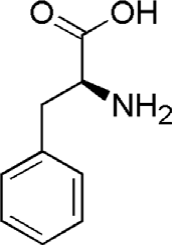
DL8	MOL001780	Tryptophan	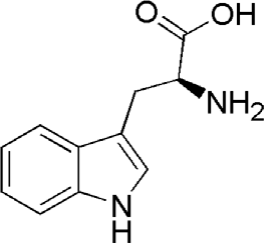
DL9	MOL001788	Adenine	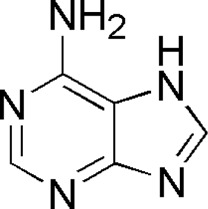
GC1	MOL004903	Liquiritin	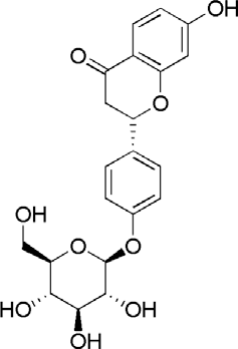
GC2	MOL001789	Isoliquiritigenin	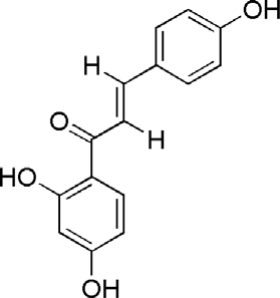
GC3	MOL004951	Isoliquiritin	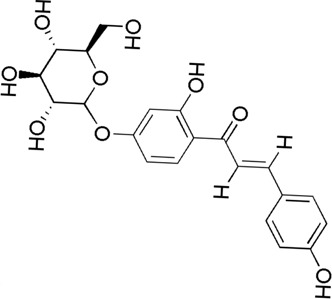
GC4	MOL004876	Glycyrrhizic acid	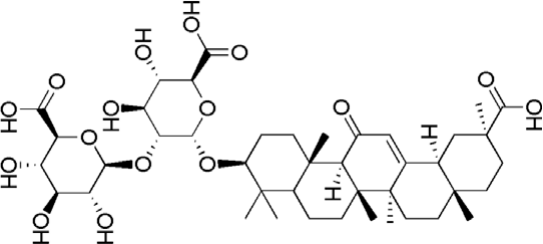
GC5	MOL000497	Licochalcone A	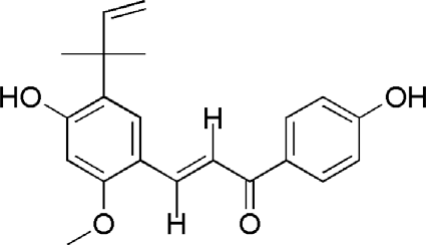
GC6	MOL004892	Licoricesaponin G2	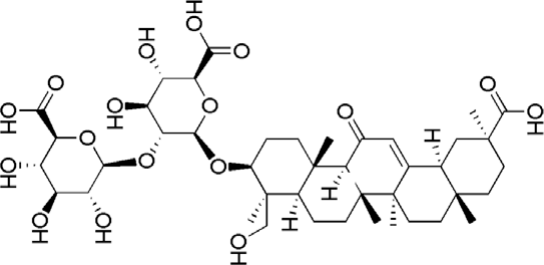
SQ1	MOL000414	Caffeic acid	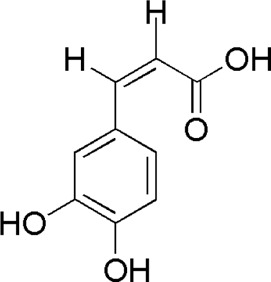
SQ2	MOL004549	3-Coumaric acid	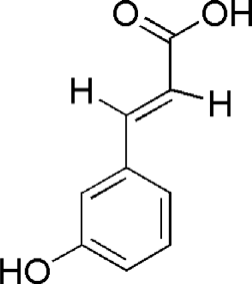
SQ3	MOL001950	Psoralen	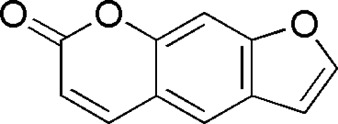
SQ4	MOL007495	Gypenoside XVII	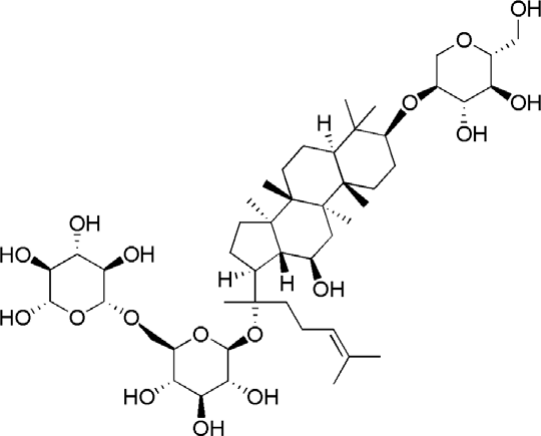
SQ5	MOL007476	Ginsenoside Rb1	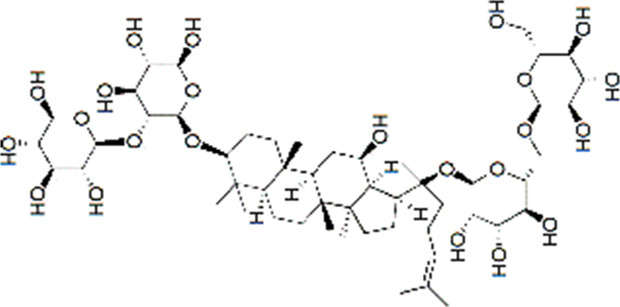
SQ6	MOL005338	Ginsenoside Re	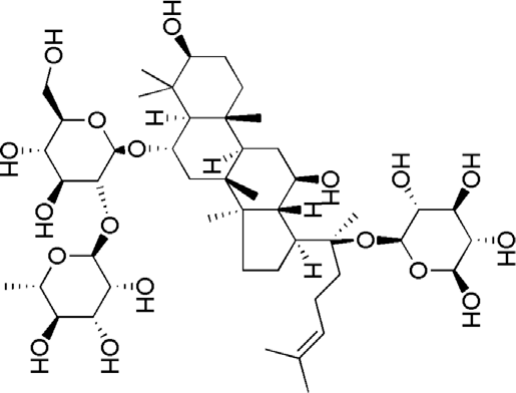

### Establishment of the ONFH Target Library

**FIGURE 3:**
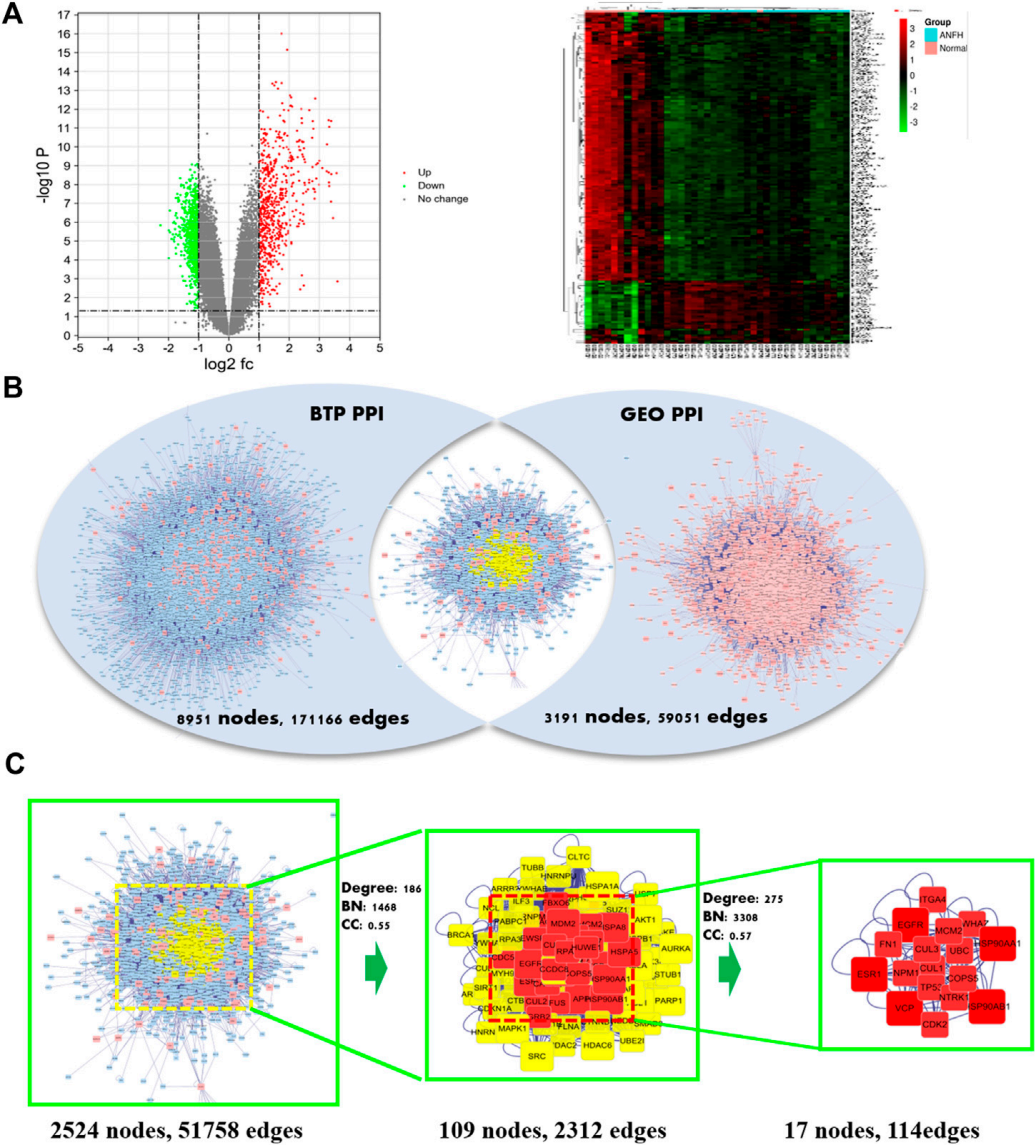
Volcano plot **(left)** and heatmap **(right)** of differentially expressed genes (DEGs) in microarray data sets of GSE123568 **(A)**. Intersection PPI network of BTP and DEGs. **(B)** Identification process of core targets of BTP against ONFH. Nodes for genes and edges for interactions between proteins **(C)**.

A total of 178 DEGs were screened out, including 143 upregulated genes and 35 downregulated genes, and were visualized with a volcano plot, as shown in Figure 2. In the volcano plot, the vertical axis represents log_2_ FC (fold change), and the horizontal axis represents log_10_ (*p*-value). The DEGs with log_2_ FC > 0 were defined as upregulated genes, and the others were downregulated genes. Additionally, the expression situation regarding all the DEGs was visualized with a heatmap, as shown in Figure 3A. In the heatmap, the vertical axis represents DEGs, while the horizontal axis represents the sample. DEGs were effectively divided into normal and ONFH groups. Red indicates the upregulated gene, and green represents the downregulated genes.

### Acquisition of Core Genes Regarding BTP Against ONFH

Centrality expresses the degree of a node in a certain network in the center of the whole network. The degree is defined as the sum of direct connections between nodes and represents the cohesion of a given node in a certain network. Furthermore, betweenness indicates the number of shortest paths passing through a node. These three factors are the indispensable basis for assessing the importance of a specific target. Through screening, 17 core targets with 114 vital interactions were obtained, and the detailed information and identification process of core targets are shown in Table 3 and Figure 3C, respectively.

**TABLE 3:** Detailed attribute values of core targets.

Name	Degree	Betweenness	Closeness
TP53	417	8838.351	0.625
EGFR	413	4978.824	0.584532
ESR1	362	4732.667	0.595238
YWHAZ	354	5282.821	0.602968
FN1	401	5764.932	0.616114
UBC	421	7284.354	0.608614
NTRK1	715	15501.89	0.680628
CUL3	519	8497.666	0.643564
CUL1	331	4143.496	0.604651
CDK2	352	5856.056	0.605778
COPS5	364	4224.348	0.605214
HSP90AB1	304	3368.986	0.577265
ITGA4	298	3802.689	0.592525
HSP90AA1	368	3823.474	0.587703
VCP	301	3662.213	0.579839
NPM1	291	3407.6	0.590909
MCM2	403	6534.106	0.617871

### Functional Enrichment of Core Targets

**FIGURE 4:**
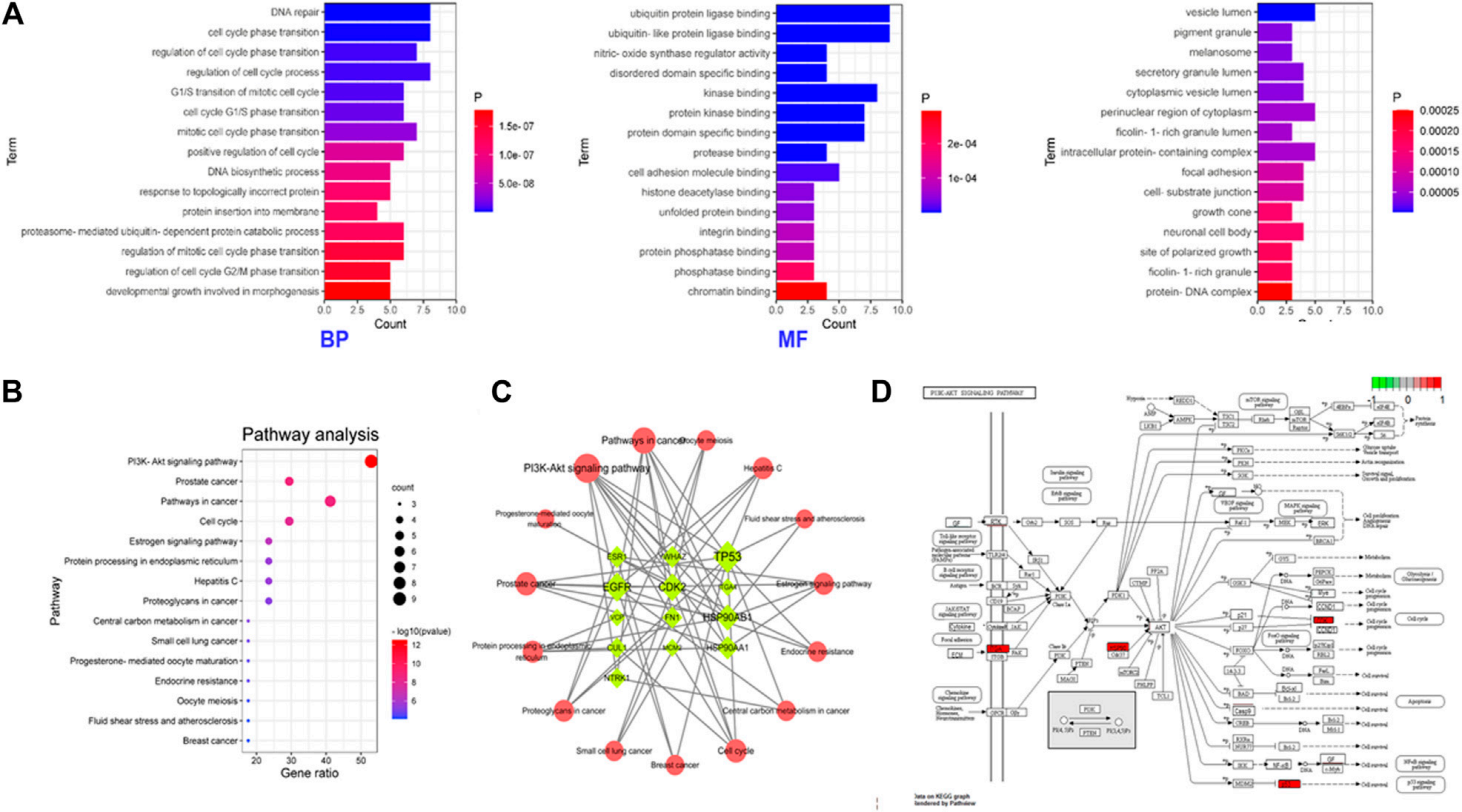
Functional enrichment of the core targets of BTP-DEGs. **(A)** GO enrichment. **(B)** KEGG enrichment. **(C)** Network of Key Targets-Pathway. **(D)** Pathway map of key targets.

The signal pathway of core targets was analyzed using the Metascape platform, and the results were visualized by bioinformatics. The results showed that the functions of core targets were closely related to the occurrence of ONFH. KEGG and GO enrichment are presented in Table 4 and Figure 4, respectively.

**TABLE 4:** Functional enrichment of the core targets of BTP-DEGs.

Term	Category	Count	*p* Value	Gene ratio
KEGG	PI3K-Akt signaling pathway	9	2.982E-13	52.94117647
	Prostate cancer	5	1.505E-09	29.41176471
	Pathways in cancer	7	1.745E-09	41.17647059
	Cell cycle	5	9.05E-09	29.41176471
	Estrogen signaling pathway	4	3.169E-07	23.52941176
	Protein processing in endoplasmic reticulum	4	2.61E-06	23.52941176
	Hepatitis C	4	2.802E-06	23.52941176
	Proteoglycans in cancer	4	5.796E-06	23.52941176
	Central carbon metabolism in cancer	3	7.797E-06	17.64705882
	Small cell lung cancer	3	1.689E-05	17.64705882
	Progesterone-mediated oocyte maturation	3	2.521E-05	17.64705882
	Endocrine resistance	3	2.521E-05	17.64705882
	Oocyte meiosis	3	5.416E-05	17.64705882
	Fluid shear stress and atherosclerosis	3	8.104E-05	17.64705882
	Breast cancer	3	8.447E-05	17.64705882
GO BP	DNA repair	8	5.631E-10	
	cell cycle phase transition	8	1.399E-09	
	regulation of cell cycle phase transition	7	6.522E-09	
	regulation of the cell cycle process	8	7.122E-09	
	G1/S transition of the mitotic cell cycle	6	9.284E-09	
	cell cycle G1/S phase transition	6	1.498E-08	
	mitotic cell cycle phase transition	7	2.936E-08	
	positive regulation of the cell cycle	6	7.962E-08	
	DNA biosynthetic process	5	9.436E-08	
	response to the topologically incorrect protein	5	1.149E-07	
	protein insertion into the membrane	4	1.196E-07	
	proteasome-mediated ubiquitin-dependent protein catabolic process	6	1.243E-07	
	regulation of mitotic cell cycle phase transition	6	1.484E-07	
	regulation of cell cycle G2/M phase transition	5	1.558E-07	
	developmental growth involved in morphogenesis	5	1.782E-07	
GO MF	ubiquitin protein ligase binding	9	2.855E-14	
	ubiquitin-like protein ligase binding	9	5.039E-14	
	nitric-oxide synthase regulator activity	4	1.142E-11	
	disordered domain specific binding	4	4.166E-09	
	Kinase binding	8	4.315E-09	
	protein kinase binding	7	5.903E-08	
	protein domain specific binding	7	8.076E-08	
	Protease binding	4	1.015E-06	
	cell adhesion molecule binding	5	1.313E-05	
	histone deacetylase binding	3	4.105E-05	
	unfolded protein binding	3	4.912E-05	
	integrin binding	3	8.104E-05	
	protein phosphatase binding	3	9.162E-05	
	Phosphatase binding	3	0.0002008	
	chromatin binding	4	0.0002956	
GO CC	vesicle lumen	5	1.147E-06	
	melanosome	3	3.392E-05	
	pigment granule	3	3.392E-05	
	secretory granule lumen	4	3.55E-05	
	cytoplasmic vesicle lumen	4	3.725E-05	
	perinuclear region of the cytoplasm	5	5.163E-05	
	ficolin-1-rich granule lumen	3	5.416E-05	
	intracellular protein-containing complex	5	5.621E-05	
	focal adhesion	4	9.86E-05	
	cell-substrate junction	4	0.0001051	
	growth cone	3	0.0001582	
	neuronal cell body	4	0.0001585	
	site of polarized growth	3	0.0001744	
	ficolin-1-rich granule	3	0.0001772	
	protein-DNA complex	3	0.0002502	

Based on the results of KEGG enrichment, as shown in Table 4, multiple pathways jointly contributed to the antiONFH effect of BTP, including the PI3k-Akt signaling pathway, prostate cancer, pathways in cancer, the cell cycle, the estrogen signaling pathway, protein processing in the endoplasmic reticulum, hepatitis c, proteoglycans in cancer, the central carbon metabolism in cancer, small-cell lung cancer, progesterone-mediated oocyte maturation, endocrine resistance, oocyte meiosis, fluid shear stress and atherosclerosis, and breast cancer. Thus, it suggested that the antiONFH effect of BTP might associate with a synergistic effect of multiple pathways.

In conformity with the results of GO enrichment, the biological processes (BPs) in which BTP chiefly participated involved DNA repair, cell cycle phase transition, regulation of cell cycle phase transition, regulation of the cell cycle process, mitotic cell cycle G1/S transition, cell cycle G1/S phase transition, mitotic cell cycle phase transition, positive regulation of the cell cycle, the DNA biosynthetic process, response to topologically incorrect protein, protein insertion into the membrane, the proteasome-mediated ubiquitin-dependent protein catabolic process, regulation of mitotic cell cycle phase transition, regulation of cell cycle G2/M phase transition, and developmental growth involved in morphogenesis.

Such effects were primarily reflected in several aspects, including ubiquitin-protein ligase binding, ubiquitin-like protein ligase binding, nitric-oxide synthase regulator activity, disordered domain specific binding, kinase binding, protein kinase binding, protein domain specific binding, protease binding, cell adhesion molecule binding, histone deacetylase binding, unfolded protein binding, integrin binding, protein phosphatase binding, phosphatase binding, and chromatin binding. The targets of this action were chiefly concentrated in multiple cell sites, including vesicle lumen, melanosomes, pigment granules, secretory granule lumen, cytoplasmic vesicle lumen, the perinuclear region of the cytoplasm, ficolin-1-rich granule lumen, the intracellular protein-containing complex, focal adhesion, the cell–substrate junction, growth cones, neuronal cell bodies, sites of polarized growth, ficolin-1-rich granules, and the protein-DNA complex.

Based on the above bioinformatics analysis, previous reports, and our preliminary experiments, we deduced that the molecular mechanisms of this TCM formula might attribute to PI3K/Akt-related cell apoptosis.

### Construction of Key Targets-Pathway Network

The network of key targets-pathway was constructed with Cytoscape (3.7.2), as presented in Figure 3B. V represents the pathway, and the diamond represents the DEGs. The greater the significance, the larger the volume of the graph was. The results showed that the targets of TP53, EGFR, and CDK2 and the pathways of the PI3K-AKT signaling pathway and cell cycle were more critical in the entire network. Therefore, subsequent experiments chose these two pathways for verification.

### Cytotoxic Effect of BTP on RAW264.7 Cells

**FIGURE 5:**
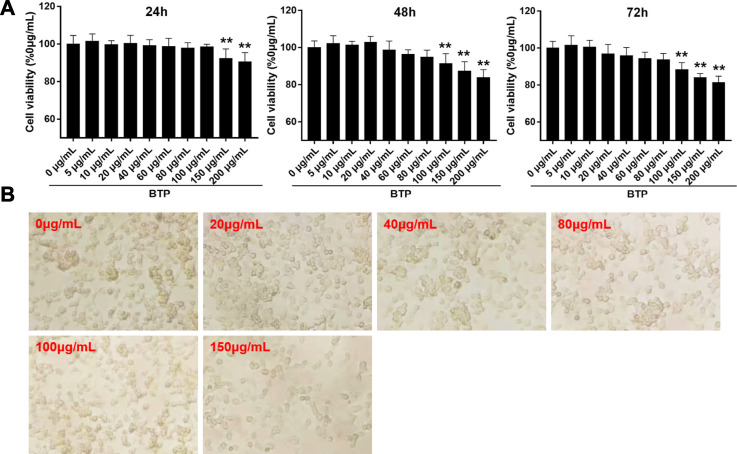
Cytotoxic effect of BTP on RAW264.7 cells. **(A)** Cell viability assays of RAW 264.7 cells treated with series doses of BTP at 24, 48, and 72 h, respectively. **(B)** Representative figures of RAW264.7 cells treated with series doses of BTP for 24 h.

The cytotoxicity of BTP on normal RAW 264.7 cells was assessed by CCK-8 assays. As shown in Figure 5, BTP at concentrations up to 200 mg/ml did not exhibit obvious cytotoxicity on RAW264.7 cells within 24 and 48 h of incubation.

### BTP Inhibits Osteoclastogenesis and Bone Resorption in Osteoclasts

**FIGURE 6:**
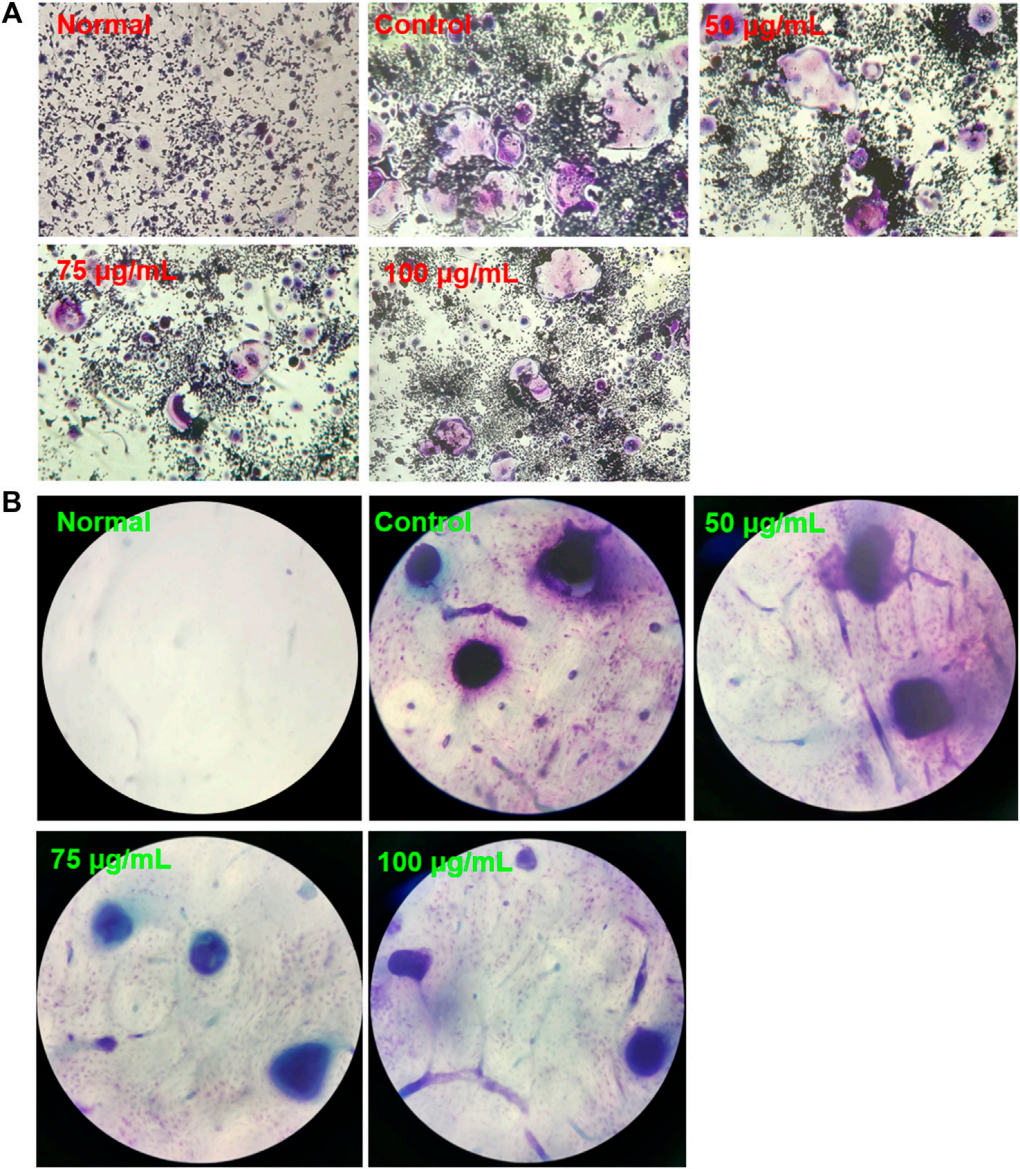
Osteoclastogenesis with TRAP staining **(A)** and bone resorption assay **(B)**.

At 7 days after inducting RAW 264.7 cells with RANKL (100 ng/ml), TRAP-positive multinucleated cells were observed under a light microscope, as shown in Figure 6. Interestingly, BTP at 20, 40, and 80 μg/ml markedly decreased the number of osteoclasts in a dose-dependent manner (*p* < 0.05 at 100 μg/ml, *p* < 0.005 at 200 μg/ml, *p* < 0.0001 at 400 μg/ml). In addition, the significant inhibitory effect of BTP on bone resorption *in vitro* has also been confirmed. As shown in Figure 8, compared with the control group, BTP dose-dependently reduced the area of bone resorption.

### BTP Induces Cell Apoptosis in Osteoclasts

**FIGURE 7:**
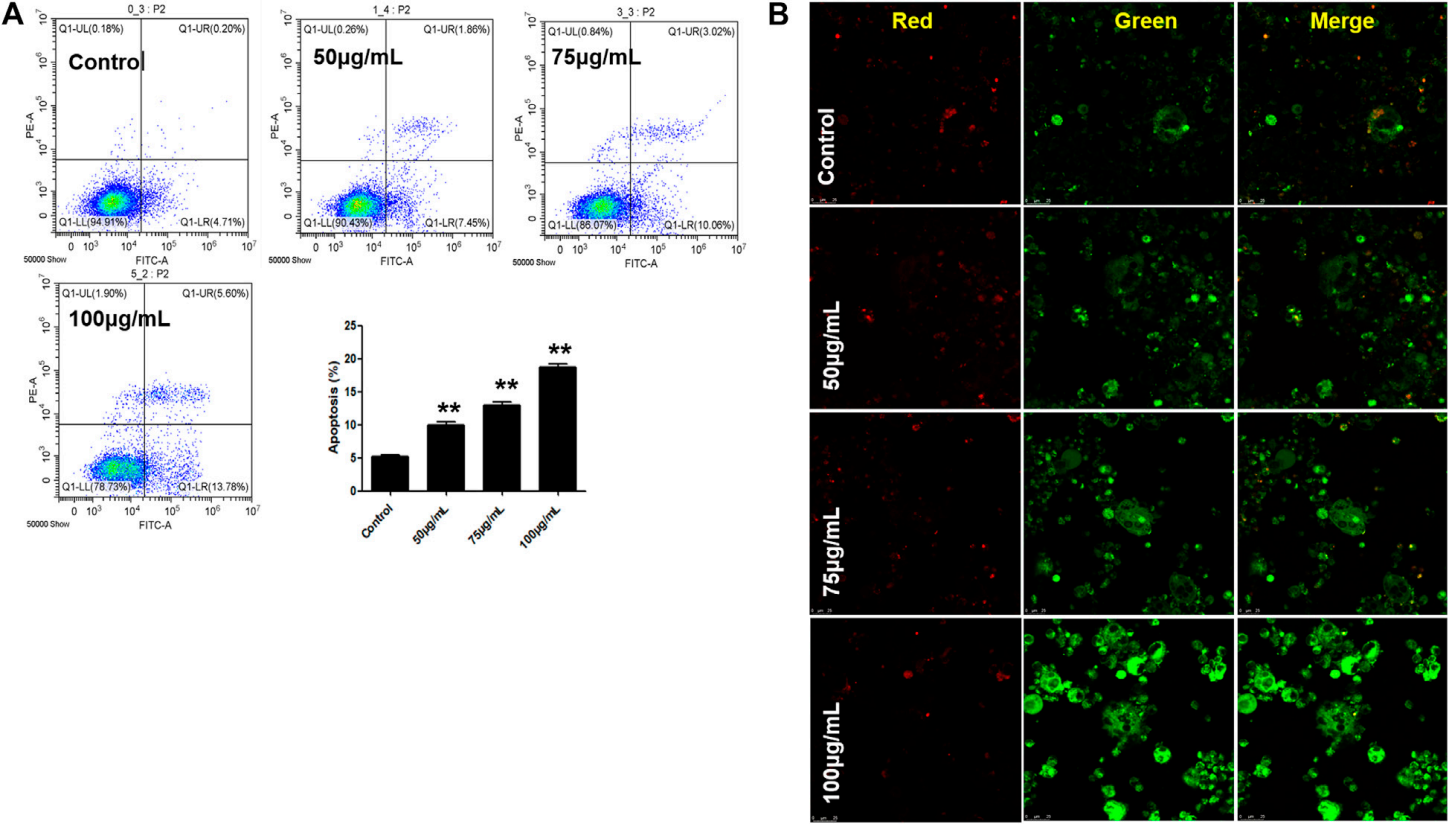
BTP induces cell apoptosis **(A)** and decreases MMP **(B)** in osteoclasts (× 400).

In this study, two comprehensively recognized cell apoptosis detection methods, including AO-EB double staining and FITC-conjugated Annexin V/PI staining, were carried out using a laser confocal microscope and flow cytometry analysis. Therefore, we further explored the apoptosis induced by BTP treatments. As shown in Figure 7A, our present results confirmed our hypothesis. After 5 days of RANKL treatment, the RAW 264.7 cells were induced to osteoclasts. As shown in Figure 7A, BTP at the concentrations of 50, 75, and 100 μg/ml could induce apoptosis in osteoclasts, compared to the control osteoclasts. Moreover, the results of immunofluorescence also presented the same trend. As far as we know, JC-1 aggregates in the mitochondrial matrix to form polymers that emit red fluorescence under normal physiological conditions and green fluorescence when MMP is reduced. Thus, the changes of ΔΨm could be directly reflected by the fluorescence transformation. In Figure 7B, compared with the control group, the green fluorescence of the experimental group was getting stronger, while the red fluorescence was getting weaker, indicating that MMP breakdown happened.

**FIGURE 8:**
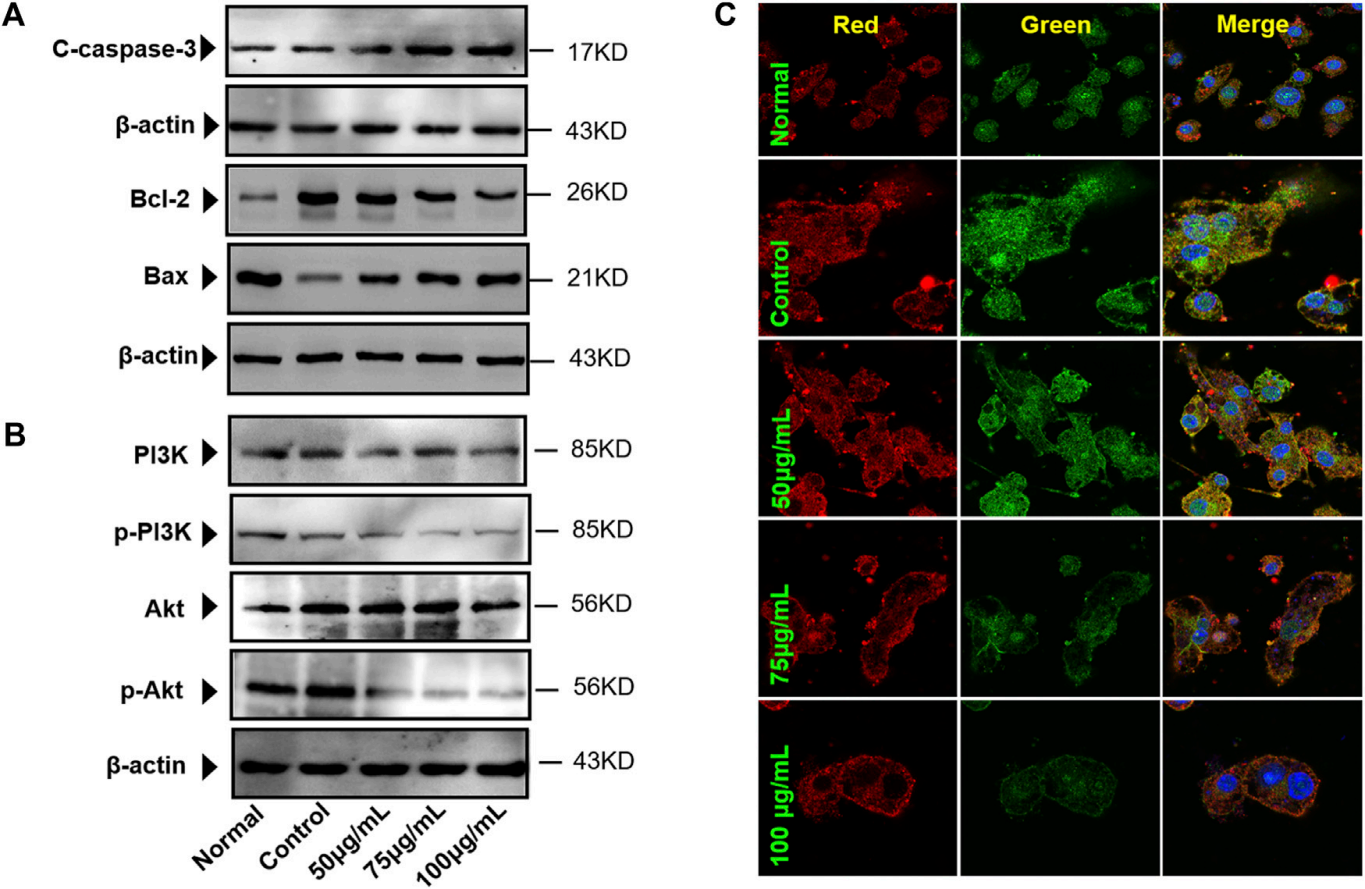
BTP regulates apoptotic proteins in osteoclasts. **(A)** Caspase-3, Bcl-2, and Bax **(B)** Akt and PI3K in osteoclasts. **(C)** Akt/p-Akt expressions in cells (× 400), p-Akt (green) and Akt (red).

The results suggested that BTP (50, 75, and 100 μg/ml) could induce remarkable apoptosis in osteoclasts (RANKL induced RAW 264.7 cells). Furthermore, the apoptosis markers of C-caspase-3, bcl-2, and bax were determined. As shown in Figure 8, BTP could upregulate the expression of C-caspase three and bax, downregulating bcl-2 expression in osteoclasts in a dose-dependent manner.

### BTP Regulates the PI3K/AKT Signal Pathway in Osteoclasts

The PI3K/Akt pathway is a classical signaling pathway responsible for regulating cell proliferation, differentiation, apoptosis, and a series of critical physiological activities. Nevertheless, only the phosphorylation of PI3K and Akt mainly participate in these physiological activities. Besides, growing numbers of studies have found that cell apoptosis is closely related to the PI3K/Akt pathway (Xue and Feng, 2018; Zhan and Yan, 2020). Therefore, we evaluated the effect of BTP on the PI3K/Akt pathway in osteoclasts. In the present study, both immunofluorescence and western blot assays were performed to determine the effect of BTP on the PI3K/Akt signaling pathway in osteoclasts. The results revealed that after pretreatment with BTP, the expression of p-Akt and p-PI3K was reduced, while no noticeable change was evident in the expression of AKT and PI3K compared to the control group (Figure 8B). Likewise, as presented in Figure 8C, the result of the immunofluorescence revealed that green fluorescence becomes weaker, indicating that the expression of p-Akt was reduced, and the red fluorescence (the expression of Akt) had no apparent change.

## Discussion

It is widely known that TCM has shown broad application prospects in treating various intractable diseases and has become increasingly recognized globally (Zhang et al., 2021; Long et al., 2020). TCM also provides abundant resources for the development of new drugs. However, it remains a challenge to study the mechanisms of action of the TCM formula due to their characteristic of multicomponents, multitargets, and multipathways (Yu and Qi, 2020). Network pharmacology, first put forward by Hopkins in 2007, can analyze the connection of components, targets, and diseases in the network systematically and study the multilayer and multipathway mechanism of action of medications comprehensively, which coincides with the fundamental concept of holism in TCM (Hopkins, 2007; Sheng et al., 2020). Thus, it has become increasingly indispensable for performing qualified basic research of TCM formulas (Zhang et al., 2021). In addition, network pharmacology may realize “drug relocation” and explore new biological activities of drugs, thus expanding the application scope. As a result, it has been widely used in pharmacological research regarding TCM.

BTP has a long history of clinical applications for ONFH treatment and is composed of *Angelica sinensis* (Oliv.) Diels, *Pheretima aspergillum* (E.Perrier), *Panax notoginseng* (Burk.) F. H. Chen, *Astragalus membranaceus* (Fisch.) Bge, and *Glycyrrhiza uralensis* Fisch. This TCM formula has the effects of nourishing blood, promoting blood circulation, dredging collaterals, and relieving pain. Although the anti-ONFH effect of BTP has been well documented, its active components and mechanisms of action remain obscure and need further research. In this study, a total of 34 compounds were identified. Among these, six compounds (caffeic acid, pimpinellin, Z-ligustilide, ferulic acid, chlorogenic acid, and benzoic acid) belonged to *Angelica sinensis* (Oliv.) Diels, seven (ononin, biochanin a, formononetin, sucrose, calycosin-7-O-β-D-glucoside, asparagine, and daidzein) to *Astragalus membranaceus* (Fisch.)Bge, six (liquiritin, isoliquiritigenin, isoliquiritin, glycyrrhizic acid, licochalcone A, and licoricesaponin g2) to *Glycyrrhiza uralensis* Fisch., eight (caffeic acid, 3-coumaric acid, psoralen, gypenoside xvii, ginsenoside Rb1, and ginsenoside re) to *Panax notoginseng* (Burk.), and nine (succinic acid, hypoxanthine, dl-arginine, nicotinic acid, guanine, guanosine, phenylalanine, tryptophan, and inosine) to *Pheretima aspergillum* (E. Perrier). Formononetin and daidzein have been reported to inhibit RANKL-induced osteoclast differentiation and regulate osteogenic differentiation, indicating that they may become potential drugs for preventing and treating bone destruction in ONFH. In addition, another study has shown that the extract of *Panax notoginseng* (Burk.) could significantly promote the repair of the femoral head in rabbits with ONFH and improve the bone density and bone microstructure (Gui et al., 2019), which was also supported by clinical research (Jia et al., 2011).

KEGG is one of the most used tools to predict the possible molecular mechanisms of drugs, particularly herbal medicines. In our present study, the KEGG enrichment of the core target genes revealed that these genes were significantly enriched in the PI3K-AKT signaling pathway and cell cycle. Numerous studies have confirmed that cell apoptosis is often accompanied by growth arrest, suggesting that cell cycle arrest correlates with apoptosis closely (Duan et al., 2021; Zhang et al., 2021). When CAF and theobromine, which can shorten the G2/M block time, were added before the exposure, the ratio of the G2/M phase decreased with more noticeable increases in apoptosis. In contrast, when TPA, IBMX, and 3-aminoben extending the G2/M block time were added, the G2/M ratio increased, while the apoptosis ratio decreased (Zhao et al., 1999). Shinomiya *et al.* used CAF to reduce the G2 blockade of mouse lymphoma cells EL-4 caused by *cis*-diamminedichloroplatinum and promote the occurrence of apoptosis (Shinomiya et al., 1997). As a result, we designed a series of *in vitro* experiments to verify our hypothesis. In the present study, CCK-8 results showed that BTP had no toxicity to RAW 264.7 cells under 150 μg/ml for 24 h treatment.

Therefore, we applied the concentration of 50, 75, and 100 μg/ml to the follow-up experiments. TRAP staining results showed that BTP at 75 and 100 μg/ml inhibited the formation of TRAP-positive multinucleated cells. In addition, the area of bone resorption *in vitro* was also significantly suppressed, illustrating that BTP could inhibit the formation of osteoclasts. In addition, the results of flow cytometry revealed that BTP could induce remarkable apoptosis in osteoclasts (RANKL-induced RAW 264.7 cells) and decrease the mitochondrial membrane potential (MMOP, ΔΨ). Consequently, we determined the apoptosis-related proteins of C-caspase-3, Bax, and Bcl-2, and found that BTP could upregulate the proapoptosis proteins of C-caspase-3 and Bax but downregulate the antiapoptosis protein of Bcl-2, which is consistent with the flow cytometry and MMOP analysis. The PI3K-Akt signaling pathway is closely related to cell apoptosis and has been recognized as a potential target for regulating cell survival and apoptosis (Zhang et al., 2019). Therefore, we speculated that the treatment effects of BTP on ONFH might associate with PI3K/Akt-mediated apoptosis. In our bioinformatics analysis, the results indicated that the effects of BTP might be closely related to the PI3K/Akt signaling pathway, which is also an important upstream signaling for apoptosis. Therefore, we determined the expressions of PI3K/Akt signal-related proteins, including PI3K, phosphorylation (p)-PI3K, Akt, and p-Akt. Our results demonstrated that BTP treatment could downregulate the expressions of p-PI3K and p-Akt, subsequently resulting in cell apoptosis of osteoclasts (RANKL induced RAW 264.7 cells).

## Conclusion

In conclusion, our present results suggested that BTP treatment could be beneficial for the osteonecrosis of the femoral head (ONFH), and the possible mechanisms are related to suppressing the formation of osteoclasts and induction of apoptosis in osteoclasts via regulation of the PI3K/Akt signaling pathway (Calder et al., 2004, Dhomen et al., 2012, Huang et al., 2004, Hua, 2017, Kimura et al., 2006, Lanaya et al., 2014, Linder et al., 2018, Lv et al., 2021, Mettey et al., 2003, Okuma et al., 2008, Poeta et al., 2007, Roovers et al., 2007, Ren et al., 2013, Savio et al., 2014, Schneider et al., 2009, Soussi, 2000, Xu et al., 2020, Yu et al., 2020, Zhang et al., 2014).

## Data Availability

The datasets presented in this study can be found in online repositories. The names of the repository/repositories and accession number(s) can be found below: PRIDE (Proteomics Identification Database, https://www.ebi.ac.uk/pride/archive). The Project Name: Analysis of components of Buxuetongluo Pills by LC-MS/MS, Project accession: PXD027269.
